# "Surface epithelial changes" in uterine endometrioid carcinoma mimicking micropapillary serous borderline tumor of ovary: report of two cases and review of the literature

**DOI:** 10.1186/1746-1596-6-13

**Published:** 2011-01-27

**Authors:** Kamaljeet Singh, Rochelle A Simon, Cunxian Zhang, M Ruhul Quddus

**Affiliations:** 1 Department of Pathology, Rhode Island Hospital, 593 Eddy Street, APC 12, Providence, RI, 02903, USA; 2 Department of Pathology, Women & Infants Hospital of Rhode Island. The Alpert Medical School of Brown University, 101 Dudley St, Providence, RI 02905, USA

## Abstract

We encountered two cases of endometrioid carcinoma of uterus with extensive surface epithelial changes (SECs) mimicking serous borderline tumor (SBT) of the ovary. The first case was a well-differentiated endometrioid carcinoma arising in a background of complex atypical hyperplasia. The second case was moderately-differentiated endometrioid carcinoma with squamous and mucinous differentiation. The SECs comprised of thin microapapillae without hierarchal branching, lined by cuboidal cells with eosinophilic cytoplasm and mild to moderate nuclear atypia. These areas were reminiscent of SBTs of ovary, micropapillary type. This report expands the existing spectrum of SECs. Serous borderline tumor of ovary like surface epithelial changes could be misleading if present in an endometrial biopsy or curettings. Therefore, knowledge of this morphologic variation is important.

## Introduction

The surface epithelial changes (SECs), typically seen in well to moderately differentiated endometrioid carcinoma of endometrium, are histologically defined by better cellular differentiation and an architectural pattern different from underlying carcinoma. The SECs can occur as microglandular, solid syncytial or as mixture of both patterns [1]. The microglandular pattern is characterized by small, tightly packed round glands, lined by cuboidal to columnar cells containing abundant eosinophilic cytoplasm with round to oval nuclei, mimicking microglandular endocervical hyperplasia. The solid syncytial type pattern shows syncytial aggregates of cells with eosinophilic cytoplasm showing micropapillary, squamoid or transitional-type differentiation. The better cellular differentiation manifests as low nuclear cytoplasmic ratio, absent or rare nucleoli and no mitosis.

The papillary syncytial metaplastic pattern is similar to papillary syncytial change seen with endometrial breakdown. We encountered two cases of endometrioid carcinoma showing exuberant SECs, histologically mimicking serous borderline tumor (SBT) of ovary. This is unusual and has not been described in literature. We discuss both cases and review associated literature of SEC.

## Case report 1

**Figure 1:**
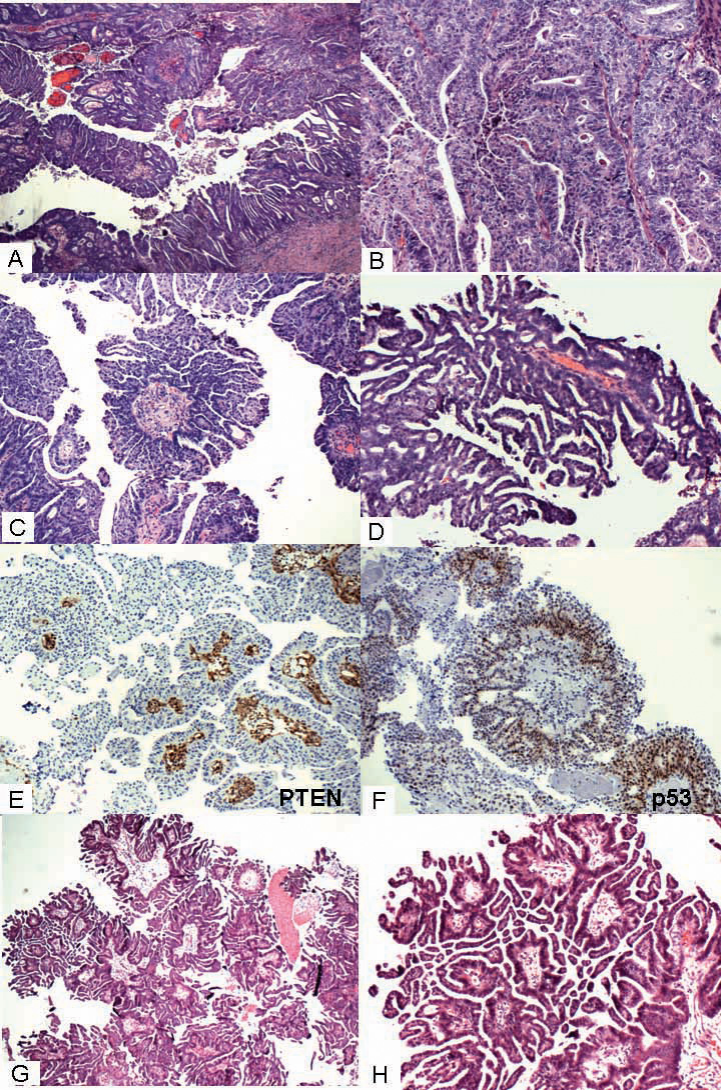
**Case 1 (A - H)**. Scanning power (A) showing well differentiated endometrioid carcinoma with SECs mimicking micropapillary serous borderline tumor of the ovary. Medium power (B) showing area of well differentiated endometrioid carcinoma, (C) with "Medusa head-like appearance", (D) multilayered cuboidal cells with "cracked" appearance. (E) PTEN-null (F) focal faint p53 immunostaining in SECs and (G, H) typical "cracked" artifact in micropapillary serous borderline tumors, low and high magnifications respectively of Case 2.

A 56 year-old female underwent total hysterectomy with bilateral salpingo-oophorectomy for uterine endometrioid carcinoma. The macroscopic examination showed a 9.0 × 5.0 × 3.0 cm polypoidal tumor involving uterine corpus and cervix invading more than 50% of myometrium. The microscopic examination showed FIGO Grade 2/3 endometrioid carcinoma with squamous and mucinous differentiation. The tumor showed exuberant SECs comprising of cuboidal tumor cells with well-defined cytoplasmic borders, moderate eosinophilic cytoplasm and round to oval nuclei with inconspicuous nucleolus. These cells were arranged in papillary pattern (Figure 1A) and classic well differentiated endometrioid adenocarcinoma (Figure 1B). This peculiar pattern was reminiscent of serous borderline tumor of ovary with "Medusa head-like appearance" (Figure 1C). Figure 1D shows multilayered cuboidal cells with "cracked" appearance. Figure 1E reveals PTEN-null tumor and Figure 1F shows focal, faint p53 immunostaining in SECs.

The uterine cervix was involved by tumor and lymphatic invasion was also identified microscopically.

## Case report 2

A 65-year-old postmenopausal female presented with chief complaint of vaginal bleeding. She underwent excision of a cervical polyp. The microscopic examination showed a well differentiated endometrioid carcinoma arising in a background of complex atypical hyperplasia of endometrium. The tumor also showed exuberant SECs, which consisted of multiple micro papillae with non-hierarchal branching, lined by cuboidal cells with well-defined cytoplasmic membrane, moderate eosinophilic cytoplasm, round to oval nucleus with inconspicuous nucleolus, reminiscent of micropapillary SBT of the ovary. Figures 1G and 1H show typical "cracked" artifact seen in serous borderline tumor of ovary, low and high magnifications respectively. These cells showed focal cytoplasmic clearing and glandular lumen formation. There was no evidence of high grade serous carcinoma.

## Discussion

In 1980, Kempson et al coined the term "endometrial epithelial metaplasias" for the phenomenon of replacement of the surface endometrial glandular epithelium with an epithelium that is normally encountered in another mullerian-derived organ such a fallopian tube or endocervix. The authors described total seven patterns of metaplasia including already well known squamous metaplasia and adding six other types including papillary, tubal, eosinophilic, mucinous, hobnail and clear cell type. Interestingly, all the 89 cases reported by Kempson et al were benign and none of them showed any evidence of invasive adenocarcinoma on hysterectomy specimen [2]. In 1987, Anderson et al reported the association of endometrial metaplasia with endometrial adenocarcinoma. Their 15/40 cases with endometrial carcinoma had associated endometrial metaplasia. The eosinophilic metaplasia was the most common metaplasia associated with endometrial cancer. An important observation made was that carcinoma occurring with metaplasia was always low-grade, occurred in young females with associated hyperplasia and hyperestrogenism[3]. A similar association between endometrial metaplasias and endometrial carcinoma was reported by Kaku T et al[4].

The metaplastic changes have been described in both benign and malignant endometrial processes. Jacques SM et al segregated the metaplastic changes in carcinomas from the benign endometrium. Jacques SM et al called the metaplastic changes occurring on the surface of the endometrial adenocarcinoma as "surface epithelial changes". They described two distinct types of patterns: papillary syncitial type and microglandular type. Most commonly, endometrial adenocarcinoma showed admixture of these two patterns, however these patterns are also present exclusively on the tumor surface. The SEC showed less atypicality than the underlying carcinoma. However, the nuclear atypia in the SEC is more than the benign endometrial metaplasia. The papillary syncitial type of SEC resembles the papillary syncitial change seen with endometrial breakdown. The microglandular type of SEC mimics the microglandular hyperplasia of the cervix[1].

The SECs mimicking the serous borderline tumor of ovary have not been reported in the literature. These SECs differ from the two types of SECs described by Jacques SM et al. In our cases, cells only focally formed glandular lumen and the cells were less atypical than the underlying tumor cells. Most of the metaplastic change comprised of thin cords of the eosinophilic cuboidal cells with distinct cell membrane arranged in micropapillae with non-hierarchal branching. In a study of 399 consecutive cases of endometrioid carcinoma, Murray SK et al identified 26 cases of uterine endometrioid carcinoma with small nonvillous papillae (ECSP). In their study, they compared ECSP with uterine serous carcinoma, and therefore did not use the term "micropapillary", to avoid confusion with micropapillary type of serous carcinoma. Murray et al concluded that endometrioid carcinoma with small nonvillous papillae may be confused with serous papillary carcinoma on microscopic examination [5].

On one hand the molecular changes in two major subtypes of endometrial cancer are well known: the estrogen-related type I shows defects in DNA-mismatch repair, mutations in PTEN, beta-catenin, and k-ras, while type II, nonendometrioid such as papillary serous and clear cell shows aneuploidy and p53 mutations [6]. The mechanism of origin of the SEC is unclear. Jacques SM proposed that SECs occur only under the condition of a larger space into which the epithelium can proliferate. Thus, SEC develops only on the endometrial surface and in the dilated malignant glands involving adenomyosis. There is a strong association of endometrial metaplasia with exogenous estrogen intake [4]. Very few studies have investigated the molecular characteristics of the SECs. Quddus et al observed weak and heterogenous p53 reactivity in the metaplastic endometrium [7]. Interestingly, p53 staining is absent in normal and hyperplastic endometrium while a strong, diffuse p53 staining is diagnostic of uterine papillary serous carcinoma. Quddus et al reported variable PTEN expression in the surface epithelial changes associated with hyperplasia and endometrioid carcinoma [8].

To our knowledge, SECs mimicking serous borderline tumor of the ovary have not been described in English literature. Our two cases expand the spectrum of the SECs described with the endometrioid carcinoma. One of the cases showed PTEN null phenotype and weak, focal p53 expression by immunohistochemistry, supporting the idea that these SECs originated from the underlying endometrioid carcinoma rather than a denovo phenomenon. Knowledge of this morphological variation in SECs may be important while interpreting the endometrial biopsy/curettage for an endometrial cancer.

## Consent

The Institutional Review Board of our academic institution does not require approval for case reports as long as no patient personal identification information or patient photograph are included. Our report does not include any personal identification of the patient or patient photograph.

## Competing interests

The authors declare that they have no competing interests.

## Authors' contributions

KS prepared the manuscript and the figures. RAS helped in preparing the final manuscript and did PTEN staining. CZ contributed one of the cases. MRQ contributed one case, initiated the case report and coordinated the preparation of the manuscript. All authors read and approved the final manuscript.
